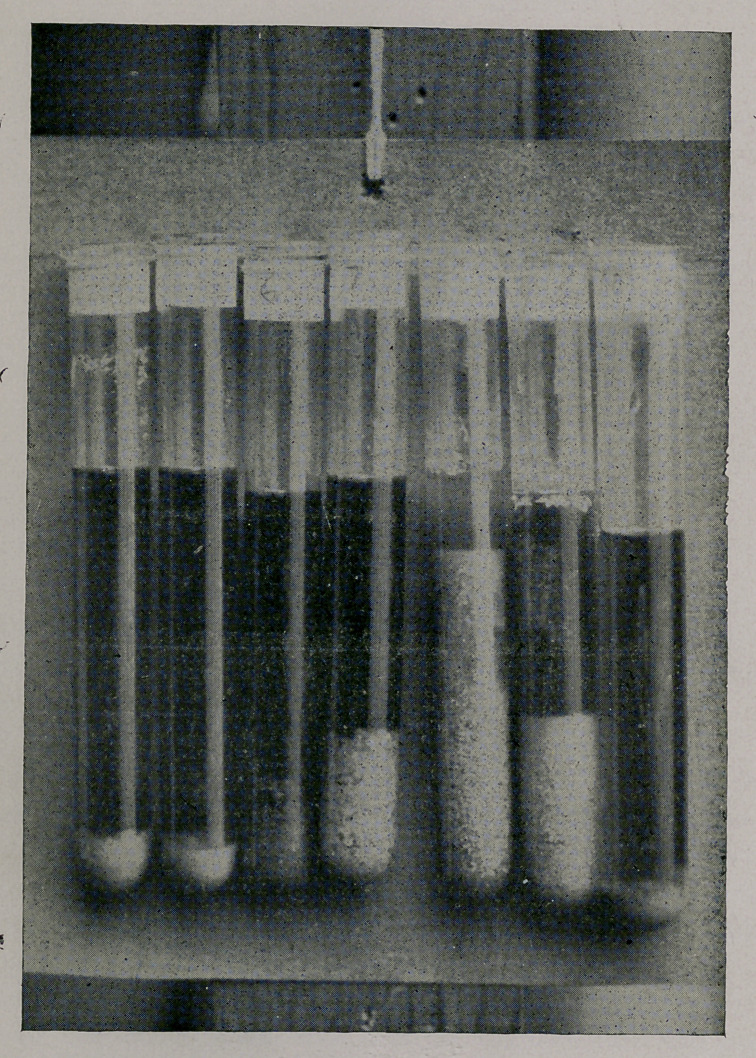# The Albuminuria of Pregnancy

**Published:** 1905-05

**Authors:** H. McHatton


### THE ALBUMINURIA OF PREGNANCY.

BY H. MCHATTON, M.D.

CASE NO. 1. White—twenty-four years—4 para. Previous labors protracted, but normal; last six years ago. Personal history neurotic. Began to suffer with digestive troubles about middle of seventh month, which gradually increased to middle of eighth month.

November 18. Urine 1023, normal.

November 19. Urine 1023, $\frac{2}{3}$ albumen.

Headache, spots before eyes, constant vomiting with rapid loss of strength. Calomel and soda ten grains each at bedtime. Saline to follow in the morning.

November 20. Urine $\frac{1}{2}$ albumen, symptoms no better. Introduced three bougies at 8 p. m. Pains began in one hour, median forceps, delivery at 3 a. m. of 21st. Urine 1010, less albumen.

November 23. Urine 1006, trace of albumen.

November 26. Urine 1018, no albumen. Maximum temperature 99$\frac{1}{2}$. Uneventful recovery.

CASE NO. 2. White—twenty-five years—primipara.

Personal history perfect, about 7$\frac{1}{2}$ months pregnant.

February 27. Headache, nausea, spots before eyes, slight edema of legs, face, neck and hands. Urine 1020, normal.

March 3. No improvement in condition. Urine 1015, 10 per cent. albumen. (The rest of the urinary record in this case is shown by photograph in days. Specimen of 6th, 1008; specimen of 8th, six hours before delivery; 9th, about eight hours afterwards.)

On 4th, 5th and 6th unpleasant symptoms increased in spite of usual treatment.

March 6. At 6 p. m. temperature 99, pulse 124, respiration 22. Two bougies inserted. Some pains by morning of the 7th, which continued in an intermittent manner. Two more bougies inserted at 6:30 p. m. Pains good but complicated by rigid os. Low forceps 6 a. m. of the 9th. During the last eight or ten hours of labor there was delirium alternating with short naps and sterterous breathing. At the time of delivery, T. 99, P. 112, R. 22.

Maxim temperature of 100 on the morning of the third day, due to retained clot. Convalescence normal.

CASE No. 3. White—twenty-two years—previous history good up to three days ago when she began to complain of severe headache, poor vision, spots before her eyes, etc. Swelling of face, eyes and legs were noticed by the family.

March 10. 5 a. m. had a convulsion. Dr. H. P. Derry was summoned, found about 40 per cent. albumen in urine. Two more convulsions before 10 a. m., when she was admitted to maternity ward of Macon Hospital. T. 96 2-10, P. 66, R. 20.

Between six and six and one-half months pregnant, os closed and rigid, complete anesthesia, manual dilatation, sufficient to admit of packing; lower segment and cervix packed with gauze. 10:45 a. m., convulsion, some pains. 6:30 p. m., convulsion, fair pains. 9 p. m., manual dilatation again. 10 p. m., high forceps, living child, which died in a few hours. 12 midnight, T. 100 2-10, P. 82, R. 26.

March 14. Albumen a trace.

March 15. None.

Temperature dropped to normal a few hours after delivery. Convalescence normal. Patient states that the previous three days to admission and fiist twenty-four hours in hospital are a complete blank to her.

CASE No. 4. White—about twenty-two years—primipara.

Seen in consultation. Nothing unusual remarked by attending physician during pregnancy. Urine normal two days before confinement, which was also normal; two hours after confinement, violent convulsion, which was repeated every thirty or forty minutes until death, which was about eight hours after first convulsion. Urine about 70 per cent. albumen.

In the first three cases there were no casts at any time. In the fourth case no examination was made of the only specimen secured.

The importance of this type of cases can best be appreciated by the fact that these four were seen in an obstetrical practice of moderate size in a period of four months. Since the practical control of puerperal fever, cases of this class are one of the most important problems of obstetrics, for on their early recognition depends the life of both mother and child.

The importance of the diagnosis of the pre-eclamptic stage is fully emphasized by the statistics of Green (Edgar's Obstetrics), which shows the maternal mortality in ante partum eclampsia to be 46 per cent., fetal mortality 69 per cent. in intra partum eclampsia, maternal mortality 25 per cent, fetal mortality 25 per cent. in post partum eclampsia, mortality of mothers 7 per cent.

In my remarks on these cases, I shall confine myself to cases of this type, and not take into consideration the 10 or 12 per cent. that occur without albumen, as I propose to take up that condition in a future paper.

The first and second of the above cases show conclusively that our urinary examinations are not made as often as they should be. Many valuable lives would be saved could we secure examinations or have the patients make some of the simpler tests themselves every two or three days after the fifth month.

It is also important in every case of pregnancy to secure a specimen of urine at the earliest possible period, and to make a complete examination of this specimen, so at a later day we will be in a position to know if we have an acute condition to deal with or an acute exacerbation of a chronic disease, as it will materially influence our prognosis. The mere recognition of albumen in moderate amount is of no material significance in many cases, as it occurs very often in conjunction with no symptoms of importance and can be easily controlled by ordinary hygienic measures. These cases demand constant watching and attention, for no one can tell when we will have an explosion. Combine the presence of albumen with the symptoms and signs of a beginning toxemia and we have a most grave condition—one that will tax our skill and judgment to the fullest extent.

Our first effort will, of course, be to control the condition by the use of medical and hygienic agencies—all the fresh air the patient can be induced to take, the observance of the best hygienic rules, and the most important single measure in this stage—an exclusive milk diet, persistent efforts to eliminate the poison through the natural outlets, bowels, kidney, liver and skin—by the usual methods and according to the indications in each individual case. If, in spite of our treatment, we find that the symptoms of the pre-eclamptic state still exist or even increase in severity, we have only one resource left, namely, empty the uterus by the least dangerous method in the given case.

When the patient has been under observation from the first, as in Cases 1 and 2 above, we can usually secure a sufficient time limit to enable us to induce labor and as a rule have no convulsions. Having decided that it is dangerous to let the pregnancy continue, my rule is to give the patient ten or fifteen grains of calomel and soda at bedtime and begin early in the morning with magnesia sulph. two-dram doses every two hours until ten or fifteen evacuations have been produced. Then have her put in as good an antiseptic condition as the surroundings will allow and under full antiseptic precaution introduce two or three solid bougies. Those known to the trade as vermilion olive pointed are the best in the market for this purpose. These should be inserted to the fundus and on different lines if possible. When they come in contact with the placenta, a partial withdrawal and reintroduction will usually overcome the difficulty.

Sometimes during the required manipulations they become too soft. A few minutes on ice will correct this condition. Care should be taken to prevent rupture of membranes, which fortunately happens rarely. The patient should then be given an anodyne and allowed to rest. If there is no indication of labor within twelve or fifteen hours, two or more bougies should be inserted in the same manner. This procedure has never failed to induce labor within a reasonable time in my experience. Should it do so, I would remove all bougies and reintroduce them, or pack the cervix and lower cavity with gauze, as seemed most appropriate in the given case. Once pains are well established, the bougies should be removed and the case allowed to progress as any normal labor—at the same time, if all indications are not most favorable, an early forcep delivery is advisable, for the longer labor lasts the more liable we are to have convulsions.

When eclampsia already exists as in the third case, if there is no effort at labor and the condition not grave, packing the lower segment of the uterus with gauze and waiting a reasonable length of time seems the safest course. Failing in this, digital dilatation and high forceps or version has thus far served me.

In fact, in these cases as in all other obstetrical work, the more assistance we can get from the natural process of labor the safer is our patient.

We can only regard the fourth case as one of those catastrophes of the toxemias of pregnancy that is liable to occur to any of us, as no indications of the grave condition were given prior to or during labor.

In following this method of practice for a good many years, I have yet to regret the induction of labor in any case, as it has been my fortune thus far not to have to record the loss of a mother or a viable child in such cases as the two above recorded.

During the same time I have regretted the non-induction of labor in some cases, and in many others where for various reasons the proper examination of the urine had not been carried out, I have seen the explosion come as from a clear sky when nothing in our art could avail to save the life of either mother or unborn child.

## Figures and Tables

**Figure f1:**